# Exploring Surgical Oncology-Based Comparative and Systematic Analysis of the Diagnostic Potential of Various Novel Biomarkers in Head and Neck Squamous Cell Carcinoma

**DOI:** 10.7759/cureus.81881

**Published:** 2025-04-08

**Authors:** Mostafa Ahmed Abdellah Ahmed, Amna Batool, Aws Khaled Mohammad Rababah, Madeeha Minhas, Abdul Mannan Jehangir, Maryum Sana, Muhammad Haseeb, Ehsan Ul Haq Mzahri

**Affiliations:** 1 General Surgery, October 6th University Hospital, 6th of October City, EGY; 2 Surgery, Fatima Memorial Hospital, Lahore, PAK; 3 Surgery, Jordan University of Science and Technology, Irbid, JOR; 4 Health Sciences, King Saud Bin Abdulaziz University for Health Sciences, Jeddah, SAU; 5 Clinical Oncology and Cancer Care, Cancer Center Nishtar Medical University and Hospital, Multan, PAK; 6 Nursing, Akhtar Saeed College of Nursing, Akhtar Saeed Medical and Dental College, Lahore, PAK; 7 Oncology, Shaikh Zayed Hospital Lahore, Lahore, PAK; 8 Pathology, University of Indonesia, Jakarta, IDN

**Keywords:** genetic biomarkers, head and neck neoplasms, squamous cell neoplasm, surgical oncology, tumor micro environment (tme)

## Abstract

Head and neck squamous cell carcinoma (HNSCC) is a widespread malignancy and has high morbidity and mortality rates. Identifying reliable biomarkers can help in improvement of diagnosis, prognosis, and treatment response. This systematic review aimed to evaluate the diagnostic potential of various novel biomarkers against HNSCC. A comprehensive search was taken place using PubMed, Scopus, Web of Science, and Google scholar that covered range of studies published from 2019 to 2025. The search focused on biomarkers that related to HNSCC and its correlation with clinical outcomes such as survival rates and treatment response. Studies were selected on the basis of predefined eligibility criteria and included observational and experimental study designs. Data extraction was performed by two independent reviewers. Study quality was assessed using the Newcastle-Ottawa Scale and the Cochrane Risk of Bias Tool for respective study designs. The quality of the evidence was evaluated using the GRADE approach. A total of 12 studies were included in this review after full screening process. The studies evaluated several biomarkers that indicated towards significant correlations between certain biomarkers and HNSCC prognosis. Elevated levels of expression of specific biomarkers were associated with poor survival outcomes, while others showed promise in the prediction of recurrence and treatment efficacy. The review highlighted the effectiveness of novel biomarkers for improving the HNSCC management. However, further validation of these findings and establish biomarkers for clinical use.

## Introduction and background

Head and neck squamous cell carcinoma (HNSCC) is one of the most widespread malignancies around the world and contributes significantly to cancer-related morbidity and mortality [1]. Even with the progress in surgical oncology, radiotherapy, and systemic treatments, survival rates are still below the ideal, especially in advanced-stage cases [2]. The primary challenge that hinders the management of HNSCC is the absence of trustworthy biomarkers for early identification, prognosis, and treatment response prediction [3]. Conventional methods rely on histopathological evaluation and imaging techniques that often fail to deliver the comprehensiveness required to study the tumor environment [4].

The identification of new biomarkers has gained acceleration recently and is offering a promising route for increasing the accuracy of diagnosis and risk assessment [5]. Immune checkpoint regulators, inflammatory mediators, cancer stem cell markers, and hypoxia-related proteins are some of the biomarkers that have come forth as possible agents for evaluating tumor aggressiveness and therapeutic responsiveness [6,7]. The biomarkers help in early detection and contribute to understanding the tumor complexity, recurrence rates, and resistance mechanisms that guide personalized treatment plans [8].

This systematic review aimed to compare the predictive value of several new biomarkers in HNSCC by focusing especially on their usefulness in surgical oncology. This review evaluated the development of molecular and immunological markers and provided insights into their clinical application and implications for enhancing patients’ experience.

## Review

Methodology

This review followed the Preferred Reporting Items for Systematic Reviews and Meta-Analyses (PRISMA) guidelines 2020 to make sure that the study is transparent and comprehensive regarding the diagnostic potential of newly available biomarkers in HNSCC. Studies were included on the basis of predetermined eligibility criteria. This criterion evaluated the study designs and focus of the study. Eligible studies contained patients who were diagnosed with HNSCC and demonstrated key clinical outcomes (survival, recurrence, and treatment response).

A search was conducted systematically by using various online databases, including PubMed, Scopus, Web of Science, and Google Scholar. The search covered studies published from 2019 to 2025 with language constraints (English only). The search strategy was designed to include keywords such as "head and neck squamous cell carcinoma", "biomarkers", "diagnosis", "prognosis", and "surgical oncology". Boolean operators were also used to combine these terms where necessary, and filters were applied to get the desired results. The search phrases used contained terms like "HNSCC biomarkers," "diagnostic biomarkers in HNSCC," "prognostic biomarkers," and "novel biomarkers for HNSCC surgery." All retrieved items were then screened for relevance to the study. The screening evaluation is based on titles, abstracts, and full texts.

Two researchers reviewed articles for eligibility based on established criteria. The reviewers resolved conflicting decisions by means of dialogue or reference to a third expert. Both reviewers conducted data extraction independently using standardized forms to collect information about study characteristics and biomarkers, along with clinical outcomes and results. Each study underwent bias risk evaluation through appropriate assessment systems, including the Newcastle-Ottawa Scale for cohort designs and the Cochrane Risk of Bias Tool for randomized controlled trials.

A narrative synthesis summarized the findings from the included studies, grouping biomarkers based on their types and relevance to clinical outcomes. No meta-analysis was performed due to study heterogeneity. The quality of evidence was assessed using the GRADE (Grading of Recommendations Assessment, Development, and Evaluation) approach, considering study design, risk of bias, and consistency of results. This methodology ensured a rigorous approach to evaluating biomarkers' potential to improve the management of HNSCC.

The narrative synthesis arranged the research findings by grouping biomarker categories to align with clinical outcomes. A meta-analysis could not be conducted because the studies exhibited substantial differences. The researchers utilized the GRADE approach to evaluate evidence quality throughout their analysis. They assessed factors such as study design, risk of bias, and results consistency to make their evaluation. The established methodology evaluated biomarkers' ability to enhance HNSCC management through systematic assessment.

Results

The review included 12 studies obtained through the screening of 92 academic articles from several electronic databases. The studies concentrated on novel biomarkers in HNSCC to assess their applications for diagnosis, prognosis, and treatment planning.

**Figure 1:**
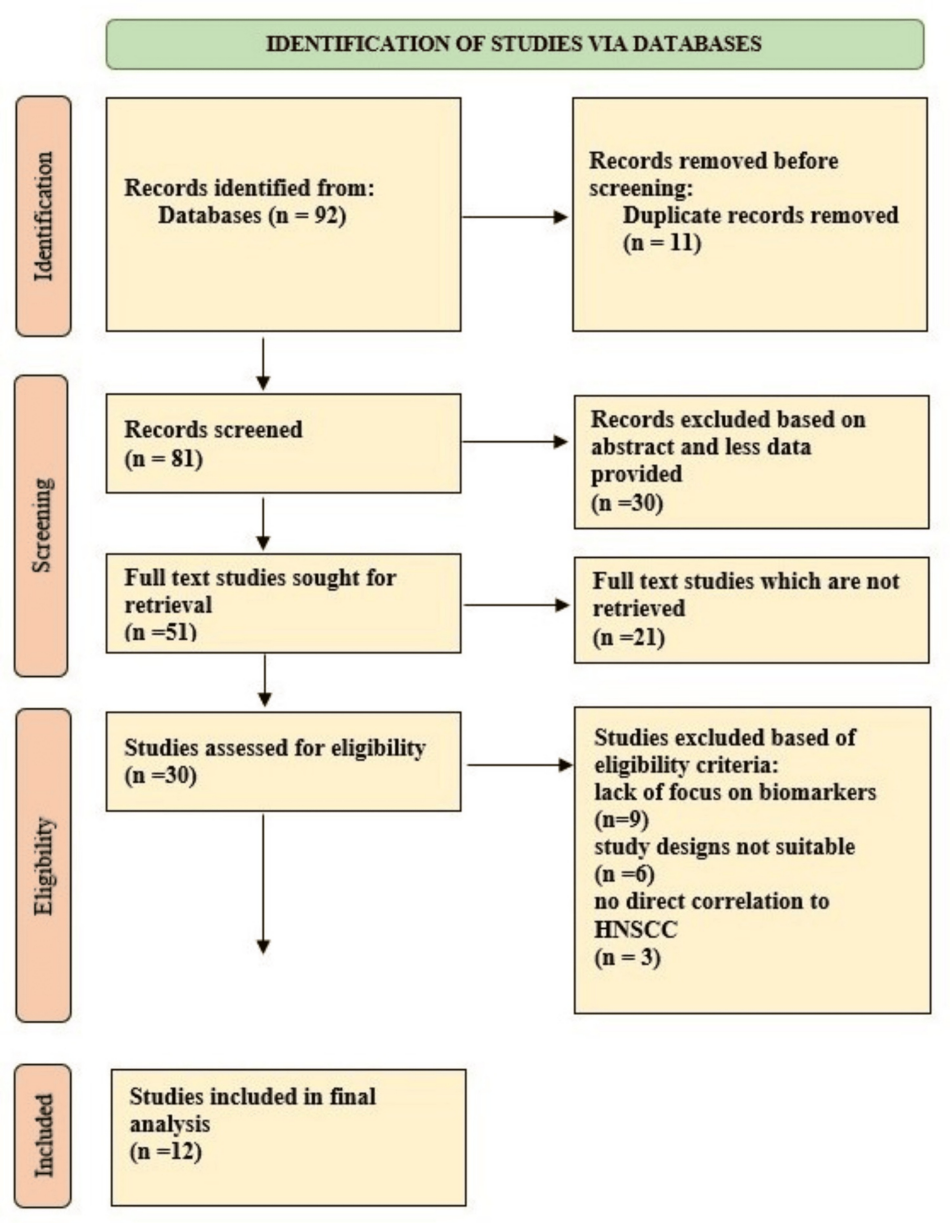
Preferred Reporting Items for Systematic Reviews and Meta-Analyses (PRISMA) flow diagram

The included studies contained various study designs, with the majority being retrospective cohort studies (n = 8), smaller numbers of case-control (n = 1), and prospective cohort studies (n = 2). The studies showcased a range of clinical outcomes, such as overall survival (OS), progression-free survival (PFS), recurrence-free survival (RFS), and treatment response. Sample sizes ranged from 36 to 488 participants, with a median of 121, and the majority of studies focused on patients with HNSCC, though some included patients with oropharyngeal, laryngeal, and esophageal cancers. Figure 1 below provides comprehensive insights into the PRISMA steps of this study.

The examined biomarkers included molecular indicators related to tumoral immune responses, cancer advancements, and therapy evasion characteristics. The screening process led to the discovery of some of these biomarkers that were not part of the original investigation scope. The combination of research correlated particular biomarkers with negative prognostic indications, such as overexpression of PD-1/PD-L1, which not only showed poor survival outcomes but also other protective effects, as shown in Table 1.

**Table 1:** Systematic review table showcasing characteristics and key findings of individual studies DSS, disease-specific survival; DFS, disease-free survival; ECOG, Eastern Cooperative Oncology Group; HPV, human papillomavirus; ICI, immune checkpoint inhibitor; LRC, locoregional control; MLR, monocyte-to-lymphocyte ratio; mTBI, modified total body irradiation; NAC, neoadjuvant chemotherapy; NLR, neutrophil-to-lymphocyte ratio; ORR, overall response rate; OS, overall survival; PET, positron emission tomography; PFS, progression-free survival; PLR, platelet-to-lymphocyte ratio; RCT, randomized controlled trial; RFS, recurrence-free survival; TNM, tumor-node-metastasis; TSCC, tongue squamous cell carcinoma

Author and Year (File Name)	Sample Size	Study Design	Confounders	Outcomes Measured	Key Findings
Huang et al., 2020 [9]	142	Retrospective cohort	Smoking, drinking, stage, PD-1/PD-L1, treatment	OS, RFS, PD-1/PD-L1	High PD-1 linked to poor OS/RFS; PD-L1 in lymphocytes was protective.
Sanada et al., 2020 [10]	43	Case series	Not assessed	Serum LOXL2, TNM, SCC antigen	LOXL2 elevated in low-grade HNSCC; needs validation.
Naruse et al., 2020 [11]	121	Retrospective cohort	Chemotherapy (NAC)	PD-1/PD-L1, recurrence, survival	PD-1/PD-L1 linked to recurrence; NAC worsened prognosis.
Lizbeth Raju et al., 2020 [12]	50	Observational	Age, grade, stage	hTERT expression	hTERT increased with malignancy; mostly cytoplasmic in OSCC.
Kato et al., 2020 [13]	70	Observational	Stage, site, recurrence	FAK/pFAK, p16, survival	High FAK/pFAK linked to recurrence and poor survival.
Elkashty et al., 2020 [14]	60	Experimental	Cell line, tumor site	CSC traits (CD44/CD271)	CD271+ cells showed resistance and high tumorigenicity.
Lee et al., 2021 [15]	125	Retrospective cohort	NLR, ECOG, HPV, size	ORR, OS, PFS	ICI response 15%; high NLR, ECOG ≥2, and large tumors predicted poor outcomes.
Dikova et al., 2021 [16]	157	Case-control	Tobacco, histology	Salivary cytokines	IL-6, IL-8, and TNF-α elevated in OSCC; linked to metastasis.
Nicolay et al., 2020 [17]	49	Prospective cohort	HPV, stage, markers	Hypoxia PET, LRC, OS	High CAIX/Ku80 linked to recurrence; HPV+ had better outcomes.
Liu et al., 2020 [18]	488	Retrospective cohort	Subsite, stage, nodes	GBP6, DSS, DFS	Low GBP6 linked to poor DSS in TSCC; potential biomarker.
Xu et al., 2022 [19]	190	RCT (Phase 2)	ECOG, age, mTBI, PD-L1	OS, PFS, biomarkers	Sintilimab improved OS; low NLR and mTBI predicted benefit.
Xun et al., 2020 [20]	151	Retrospective cohort	Age, site, surgery	OS, PFS, inflammation markers	High NLR, PLR, and MLR predicted poor survival; independent markers.

This systematic review utilizes research findings that explore survival and recurrence connections between biomarkers across HNSCC. According to Huang et al., both high PD-1 expression and PD-L1 expression in lymphocytes displayed contrasting survival patterns where PD-1 displayed adverse effects, yet PD-L1 showed protective properties [9]. Naruse et al. explored how PD-1/PD-L1 levels contributed to disease recurrence while demonstrating that neoadjuvant chemotherapy diminished treatment success according to their research [10,11]. Lizbeth Raju et al. studied human telomerase reverse transcriptase (hTERT) expression and established its correlation to malignant progression as well as its primary cytoplasmic localization pattern within oral squamous cell carcinoma (OSCC) tissues [12]. Global findings presented by Kato et al. showed that higher levels of FAK/pFAK proteins indicate a link between negative patient outcomes and HNSCC recurrence. Likewise, Elkashty et al. worked on CSC traits [13,14]. Lee and colleagues discovered that patients having a high neutrophil-to-lymphocyte ratio (NLR) and Eastern Cooperative Oncology Group (ECOG) performance status ≥2 and large tumor dimensions showed a limited immune checkpoint inhibitor (ICI) response rate of 15% in their patient cohort [15]. The research conducted by Xun et al. established that higher inflammatory markers, including NLR, platelet-to-lymphocyte ratio (PLR), and monocyte-to-lymphocyte ratio (MLR), served as independent indicators of insufficient survival outcomes [19,20]. The large study conducted by Liu et al. demonstrated that low GBP6 expression independently correlates with worse tongue squamous cell carcinoma disease-specific survival, thus establishing GBP6 as a potential biomarker [18]. Research by Dikova et al. and Nicolay et al. indicated that cytokines and biomarkers, including IL-6, IL-8, TNF-α, CAIX, and Ku80, are frequently elevated in non-HPV positive patients for recurrence or metastasis development [16,17].

The risk of bias in most studies reached either moderate or high levels. Cohort studies maintained a higher risk due to unrandomized designs, uncertain allocation procedures, and study participant selection issues. The Newcastle-Ottawa Scale showed that cohort studies had varying levels of bias control for confounding variables. The Cochrane Risk of Bias Tool showed that randomized controlled trials contained incomplete data for outcomes and uncertain blinding protocols during assessment, as shown in Table 2. Reviewer disagreements required discussion until a consensus was reached. When necessary, a third reviewer participated in resolving such conflicts.

**Table 2:** Risk of bias assessment of individual randomized clinical trials "+" indicates a low risk of bias, "±" indicates an unclear or moderate risk of bias, and"-" indicates a high risk of bias.

Study	Sequence Generation - Selection Bias	Allocation Sequence Concealment - Selection Bias	Blinding of Participants and Personnel - Performance Bias	Blinding of Outcome Assessment - Detection Bias	Incomplete Outcome Data	Selective Outcome Reporting	Other Bias
Xu et al., 2022 [19]	+	+	±	+	+	+	+

The GRADE assessment system was employed to evaluate the strength of evidence by examining research designs, risks of bias, and outcome concurrences. The evidence supporting the use of novel biomarkers for HNSCC diagnosis displayed promising results, yet received a moderate rating. Multiple research studies had limitations such as small sample size, lack of valid prospective testing, and other methodological defects. According to the GRADE assessment, most biomarkers lacked sufficient evidence to justify their direct use in clinical settings. These biomarkers demonstrated promising results, but they require additional validation through larger multi-center research investigations, as shown in Table 3.

**Table 3:** Risk of bias assessment of individual observational studies

Study	Selection (Max 4)	Comparability (Max 2)	Outcome (Max 3)	Total Score (Max 9)
Huang et al., 2020 [9]	★★★★	★★	★★★	9
Sanada et al., 2020 [10]	★★★★	★	★★	7
Naruse et al., 2020 [11]	★★★★	★	★★★★	9
Lizbeth Raju et al., 2020 [12]	★★★	★	★★	6
Kato et al., 2020 [13]	★★★★	★	★★★	8
Elkashty et al., 2020 [14]	★★★★	★	★★	8
Lee et al., 2021 [15]	★★★	★	★★	6
Dikova et al., 2021 [16]	★★★★	★	★★★	8
Nicolay et al., 2020 [17]	★★★★	★	★★★	8
Liu et al., 2020 [18]	★★★	★	★★	6
Xun et al., 2019 [20]	★★★★	★	★★	8

Discussion

The study reviewed systematically the diagnostic value of emerging biomarkers in HNSCC while investigating their influence on surgical oncology. Twelve studies were thoroughly evaluated to establish a correlation between novel biomarkers and clinical outcome measures. Biomarkers have shown promising capability to enhance HNSCC-based diagnosis along with better prognostic and therapy planning [21].

The findings of this review showcased a wide assortment of biomarkers, such as molecular signatures associated with immune response, tumor advancement, and resistance to treatment. Numerous studies confirmed that elevated expression of PD-1/PD-L1 served as an independent factor that predicted lower survival rates among HNSCC patients [22]. Current literature on other cancers supports the findings that showcase the role of immune checkpoint proteins in immune evasion, correlating with tumor aggressiveness. Research also indicates that poor prognosis occurs from elevated levels of various biomarkers linked with hypoxia, cell proliferation, and cancer stem cells [23].

The results from different biomarkers across studies highlighted the obstacles that prevent biomarker adoption in clinical settings. While some biomarkers seemed reliable, a lack of standardized protocol and insufficient large-scale prospective studies restrict the reliable application of biomarkers relating to clinical outcomes such as OS and PFS [24]. The majority of the analyzed research worked with limited sample sizes, thus creating conditions for biased results and limiting the strength of evidence. The variation in study design, including heterogeneous patient demographics as well as tumor locations and cancer stages, complicated the ability to establish generalizable results regarding biomarker suitability across distinct HNSCC patient groups.

Clinical use of several biomarkers appeared promising in some studies, but their validation across multiple centers among trials is essential. Hypoxia-inducible factor 1-alpha (HIF-1α) stood out as a promising biomarker for finding aggressive disease patterns [25]. The response to treatment and progression of the tumor were found to be linked with markers that focused on functions of immune response, like tumor-infiltrating lymphocytes and cytokine levels [26]. The identification of biomarkers that link to particular tumor actions, such as invasion and metastasis, could help doctors make treatment choices, especially in the context of personalized medicine approaches [27].

Researchers are evaluating the clinical effects that occur when ICIs focus on blocking PD-1/PD-L1 pathways in HNSCC. The evidence about outcomes of PD-1/PD-L1 expression prediction in relation to ICI response showed inconsistent results, but some studies identified higher PD-1/PD-L1 expression as a marker for poor prognosis and survival [28,29]. Tumor microenvironments, together with immune cell infiltration and treatment background, generate complex interactions that cause discrepancies among immune biomarkers in cancer outcomes. Further research on these factors, together with additional markers, is essential to enhance understanding of their predictive power [30].

Similarly, several weaknesses were seen during the evaluation of the risk of bias. The methodological flaws created moderate to high risks in studies, such as insufficient control of confounding elements, lack of randomized cohort design, and partial outcome reporting. The overall high bias risk among included studies limited the strength of evidence while potentially producing conflicting research results. Future studies should focus on the diagnostic and prognostic potential of these biomarkers to validate their clinical applications.

## Conclusions

In conclusion, this systematic review highlights the potential of novel biomarkers. Many biomarkers demonstrated promising correlations with clinical outcomes, but the overall quality of the evidence was moderate, while some studies showed a high risk of bias. The findings suggest that there should be further validation in larger, multicenter, prospective studies in order to establish the clinical utilization of these biomarkers.

The addition of these biomarkers into clinical practices would eventually enhance patient outcomes through better precision in diagnosis, prognosis, and customized treatment approaches. However, their role in HNSCC management can be studied further rigorously.
